# Lung cancer incidence among workers biologically monitored for occupational exposure to lead: a cohort study

**DOI:** 10.5271/sjweh.4046

**Published:** 2022-10-01

**Authors:** Ahti Anttila, Sanni Uuksulainen, Matti Rantanen, Markku Sallmén

**Affiliations:** 1 Finnish Cancer Registry, Helsinki, Finland; 2 Finnish Institute of Occupational Health, Helsinki, Finland; 3 Retired

**Keywords:** association, blood lead level, carcinoma of the lung, employee, lead compound, risk

## Abstract

**Objective:**

Earlier studies have reported increased risks of lung, kidney and brain cancers for exposure to lead. The International Agency for Research on Cancer (IARC) Working Group evaluated inorganic lead and its compounds probably carcinogenic to humans. This study aimed to assess the association between blood lead level in occupational exposure and risk of lung cancer.

**Methods:**

The study was based on the follow-up of lung cancer incidence during 1973–2014 among 20 729 employees biologically monitored for their occupational lead exposure in 1973–1983. Duration of employment in the monitored work was assessed using records from the Finnish Centre for Pensions; and potential confounding by other occupational carcinogens using longitudinal information on the occupation in censuses and the Finnish National Job-Exposure Matrix (FINJEM). Occupation- and gender-specific prevalence of regular tobacco smoking and the socioeconomic status were also utilized in the adjustments for potential confounding.

**Results:**

Positive trends were found for the elevated blood lead levels on the lung cancer risk. Among employees with the duration of employment of ≥60 months, the relative risk (RR) of lung cancer was 1.72 [95% confidence interval (CI) 1.28–2.31] for mean blood lead 1.0–1.9 µmol/L and RR 2.63 (95% CI 1.71–4.05) for mean blood lead ≥2.0 µmol/L, compared with mean lead <0.5 µmol/L. The studied potential confounders did not explain the findings on the increased risk for lead exposure.

**Conclusions:**

The current study lends support to the findings that exposure to lead increases lung cancer risk. Increased risks were seen already at rather low blood lead levels.

Studies on occupational exposure to inorganic lead have inconsistently reported increased risks of lung, kidney and brain cancers (1). On the other hand, increased risks were suggested at environmental exposure levels clearly lower than the occupational safety standards. In part, the excess risk within such low-dose populations could have been due to residual confounding from tobacco smoking histories. There was extensive evidence from animal experimental studies showing that lead compounds can induce kidney tumors and brain gliomas, and lead proved to be an effective renal carcinogen or tumor promoter (1). The International Agency for Research on Cancer (IARC) Working Group evaluated inorganic and its compounds probably carcinogenic to humans, Group 2A, based on sufficient evidence from animal studies and limited evidence from human studies.

It has been recommended to use blood lead cohorts in assessing human cancer risks in lead-exposed workers due to availability of reliable individual-level exposure measures (2). Since the IARC evaluation, follow-up studies of occupational cohorts with blood lead data from Australia (3), South Korea (4) and UK (5) have shown variable results. Blood lead from environmental exposures has also been further reported to correlate with all-cause, all-cancer and lung cancer mortality, adjusted for cigarettes smoked per day, alcohol consumption, poverty income ratio and several other potential confounders (6).

In Finland, a cohort of employees monitored for occupational exposure to lead has been collected (7, 8). The earlier Finnish follow-up studies of the cohort, available in the IARC evaluation, showed increased risks of lung and brain cancers (9, 10). This cohort participated in a recent multicenter cohort mortality study in three countries (USA, Finland, and UK); significant positive trends were found with increased blood level for overall mortality and for mortality from lung cancer, chronic obstructive pulmonary disease (COPD), stroke and heart disease (11). Updated cancer incidence follow-up of two cohorts (Finland and UK) documented positive associations with increasing blood lead level for several cancer types, including lung and brain cancer particularly in the Finnish material (12). Information for potential confounders was not available for the above multicenter studies.

The purpose of the current study was to assess the association between blood lead level in occupational exposure and risk of lung cancer. The study was based on the follow-up of lung cancer incidence in the Finnish cohort biologically monitored for their occupational exposure to lead, taking in account the exposure histories. Potential confounding by other occupational carcinogens were assessed using information on the occupation in censuses and the Finnish National Job-Exposure Matrix (FINJEM) utilizing longitudinal census records. Occupation- and gender-specific prevalence of regular tobacco smoking (hereafter: smoking prevalence) and the socioeconomic status (SES) were also utilized. There was an emphasis to assess risks already at rather low blood lead levels, and analyses were done using both external and internal comparison groups.

**Figure 1:**
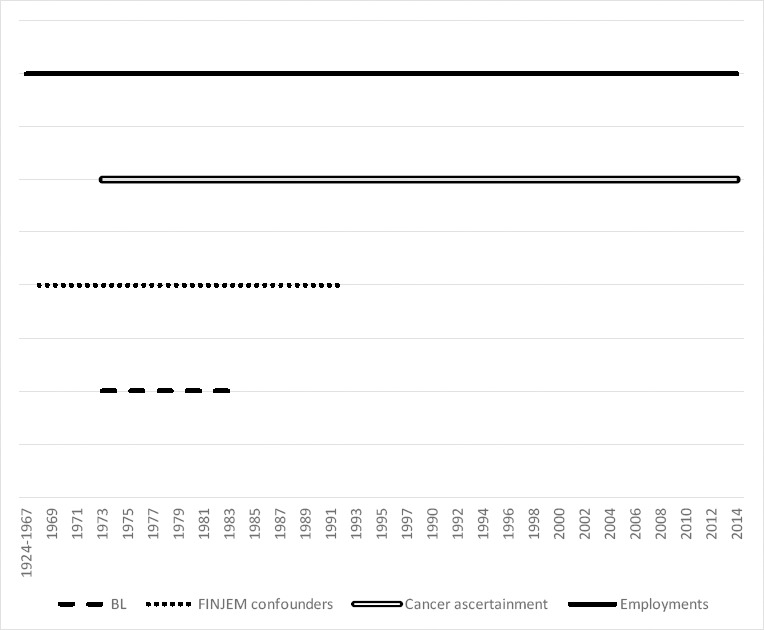
The periods during which there was information on blood lead measurements (BL), on emplyments covering BL, on confounders, and on the cancer ascertainment.

## Methods

### Study materials

The cohort includes employees with documented blood lead (BL) measurements at the Finnish Institute of Occupational Health (FIOH) in the period 1973–1983, based on laboratory reports and employer data. The employees were identified for the monitoring based by the occupational safety and occupational health organizations of the employer or initiatives of employers only. Monitoring and related exposure prevention and safeguarding were based on the legal framework for occupational safety that obliged the employer to conduct these actions. Finnish labor law mandated that if the BL of any worker in the workplace exceeded 1.9 μmol/L, then all employees in that workplace should have their BL measured. The most regular BL tests were taken from the lead battery industry, lead smelting and metal foundries (13). There were also workplaces from a variety of activities and industries, often with only few BL measurements from a workplace. The monitoring was performed also in lead-using workplaces where there was no appropriate information about the exposure levels of the employees. If the BL levels were lower than the action limits, no further follow-up was legally requested. The current study updates the cancer incidence follow-up information up to end of 2014.

The correctness of the personal identifiers was checked from the Population Information System (dvv.fi/vaestotietojarjestelma), digital and population data services agency; data checked 19 September, 2014), and the dates of potential emigration or death identified up to end of 2014. There were altogether 63 700 BL in the original data. Measurements records were excluded from the current study if (i) it was not possible trace the personal identifier, (ii) the person was <18 or >65 years at time of the measurement or (iii) the measurement was done for non-occupational reasons. There were 59 920 measurements from 20 752 employees after these exclusions. Finally, we excluded 23 persons with incorrect personal identifiers, leaving altogether 20 729 employees in the final cohort (2408 women and 18 321 men). There are on average three BL measurements per person and only one measurement for about 60% of the cohort members. About 11% of the highest personal BL were >1.9 μmol/L and about 16% were <0.5 μmol/L corresponding BL levels in the Finnish general population during the monitoring period. In 1970–1973, mean BL was 0.58 µmol/L in men and 0.46 µmol/L in women; the range was 0.1–1.6 µmol/L (14).

Incidence records of lung cancers were obtained for the cohort from 1973 up to end of 2014 from a linkage with the Finnish Cancer Registry, using the personal identifier as the key. ICD-10 codes C33 and C34 (lung, trachea or bronchus) were included in the linkage. Data on the occupation and SES were obtained from Statistics Finland data on censuses for years 1970, 1975, 1980, 1985 and 1990. In census 1970, the data on employment, main occupational activity, and education was collected from citizens of Finland. In later years, data were collected from multiple registers and other available electronic data sources. SES in 1975 was assessed in census records based on information of the main occupational activity, occupational status, education, and field of activity. Five classes were used in our analysis: (i) entrepreneurs and upper-level employees; (ii) lower-level employees; (iii) manual workers; (iv) students; and (v) unknown or missing SES. Information on employment periods for the cohort members were traced from the earnings and accrual register of the Finnish Centre for Pensions, including year 1961 or later (www.etk.fi/en/services-for-experts/registers/register-descriptions). A graph on the timelines for information on BL measurements, on confounders, and on the outcome ascertainment is provided in the figure S1 of the supplementary material (www.sjweh.fi/article/4046).

FIOH permitted the use of the monitoring data (TTL/2/2014). The linkage between the cohort and the cancer registry files was based on the permit by the Finnish Institute for Health and Welfare (THL/738/5.05.00/2014; THL/1443/5.05.00/2020). The study materials had permits also by the Digital and population data services agency (VRK 1770/410/14), Statistics Finland (TK-53-796-15; TK-53-804-20) and Finnish Centre for Pensions (ETK 27.1.2015; ETK/SUTI 19032). The study used only data from the register-based sources that were permitted by the above authorities, therefore evaluation by an ethical committee was not required.

### Exposure assessments

Exposure to lead was solely based on individual BL measurements. Both maximum and mean BL as categorized as well as continuous were used in the analysis. The length of monitored employment periods was categorized to four groups (<6, 6–59, ≥60 months, unknown), enabling identification of short-term employment as well as tentative analysis by duration. The number of personal measurements was often small and employment periods often extended outside monitoring period 1973–1983. The duration of employment was unknown if no valid employment records were available in the pensions center. There was no further information on work tasks during the monitored employment periods, restricting use of more detailed cumulative exposure indices for lead.

Altogether 11 exposures were assessed from FINJEM as potential occupational confounder for lung cancers, based on the IARC classes 1 or 2A and findings from the initial study (9): asbestos, chromium, nickel, arsenic, cadmium, quartz dust, respirable dust, gasoline engine exhaust, diesel engine exhaust, polycyclic aromatic hydrocarbons, and benzo(a)pyrene. Exposures to these chemicals were assessed on the basis of the occupational group of the employees, available in population censuses carried out every five years in 1970–1990 and FINJEM (15, 16). The FINJEM exposure assessments uses a three-digit coding structure of the occupational groups, following closely the ISCO58 classification. Exposure assessment procedure was identical for all the subjects and was blind to lung cancer outcome. First, annual mean exposure of each selected chemical was defined as a product of probability and level of exposure for the five census years. In addition to true zero values in FINJEM, annual mean exposure of all the chemicals was set to zero in censuses where (i) the study subject had no occupation or occupation was unknown or (ii) SES was student, pensioner/retiree, unemployed or unknown. In 192 subjects, occupation was missing or unknown in all the five censuses. In occupational groups exposed to any of the 11 potential occupational confounder, prevalence of any exposure in censuses 1970–1990 ranged from 14% (arsenic) to 77% (respirable dust). More detailed information on exposure to confounders by mean BL classes are presented in supplementary table S1.

To assess cumulative exposure (CE) for the potential occupational confounders, the annual mean exposure values in the five censuses were used in the 25 calendar years 1968–1992 as follows. Occupational group in each census was based on personal activity in the last week or the main occupation of the census year. Occupation in each census 1970–1990 was assumed for census year plus two calendar years. Finally, CE was the sum of mean exposures in this period. In the case of end of follow-up prior to 1 January 1993, the end year and years thereafter were omitted from cumulative exposure.

Smoking prevalence was assessed based on regular smoking data in annual health surveys in 1978–1993 (17).

### Statistical methods

Follow-up of lung cancer incidence was initiated in the standardized incidence ratios (SIR) analyses at time of the last personal measurement and closed at death, emigration, or end of the follow-up period, whichever came first. SIR of lung cancer were calculated by comparing the observed numbers in the cohort follow-up with the expected numbers of cancers by age, gender and (calendar) period based on the risks in the Finnish general population and assuming that the observed numbers followed Poisson distribution. In addition to the risk estimates by the BL categories, the P-value for a linear trend over BL categories was assessed with multivariable Poisson regression. These external comparisons were restricted to few descriptive analyses using only the BL data as the source of exposure. Internal comparisons of the lung cancer risks by BL level were done using Cox proportional hazards model (R Studio). In the internal analysis the follow-up time was closed for lung cancer cases at the diagnosis time of their first lung cancer; otherwise as in the external analysis. In the internal comparisons, the reference categories were formed based on the personal results (highest or mean BL) and the duration of the monitored employment period. For the categorical internal analyses, the data was condensed into four groups (0.0–0.4, 0.5–0.9, 1.0–1.9, and ≥2.0 µmol/L) based on the personal mean or maximum BL. BL indicators were modelled also as continuous variables using natural logarithms of BL. The models were adjusted for age, gender, SES, year of last personal measurement, and smoking prevalence. Furthermore, to test confounding due occupational exposure, continuous variables on cumulative exposure to eleven potential occupational carcinogenic FINJEM factors were used in separate models. Primarily, those occupational exposures were thought informative as a confounder, if the adjustment for it changed the RR point estimate for the highest BL group (mean personal BL ≥2.0 µmol/L) by ≥5% [see (18)]. Lung cancer risk was described also by the lead industries with most regular monitoring due to high levels (lead battery industry, lead smelting, metal foundries).

## Results

There were 644 842 person-years in the study. There was a small increase in the lung cancer risk in the whole cohort compared to the general population [SIR 1.23, 95% confidence interval (CI) 1.14–1.32; table 1]. The SIR was higher among women (1.96, CI 1.47–2.57) than men (1.19, 95% CI 1.10–1.29). In the lowest BL category – 0.0–0.4 µmol/L – the SIR was 0.81 (95% CI 0.62–1.04) and the risk increased statistically significant above unity for blood levels ≥1.0–1.4 µmol/L.

**Table 1:** Number of persons, person-time, observed and expected number of lung cancer cases and standardized lung cancer incidence ratio (SIR) with 95% confidence intervals (CI) in the cohort follow-up. **Bold indicates statistical significance**.

Analysis group	Persons	Person-years	Observed cases	Expected cases	SIR	95% CI
Whole cohort	20 729	644 842	690	561	**1.23**	**1.14–1.32**
Men	18 321	567 002	638	535	**1.19**	**1.10–1.29**
Women	2408	77 840	52	26.5	**1.96**	**1.47–2.57**
Highest personal blood lead value µmol/L, whole follow–up						
0.0–0.4	3236	100 880	64	78.8	0.81	0.62–1.04
0.5–0.9	8523	268 858	262	241	1.09	0.96–1.23
1.0–1.4	4643	145 170	169	130	**1.30**	**1.11–1.51**
1.5–1.9	1962	60 074	83	50.2	**1.65**	**1.32–2.05**
2.0–2.9	1720	51 234	75	45.1	**1.66**	**1.31–2.08**
3.0–7.8	645	18 625	37	16.4	**2.26**	**1.59–3.12**
Follow–up time since the last measurement (years)						
0–9		201 858	98	87.4	1.12	0.91–1.37
10–19		188 590	165	142	1.16	0.99–1.35
≥20		254 394	427	332	**1.29**	**1.17–1.42**

The risk increased slightly as longer follow-up times passed since the last personal measurement (table 2). In the shortest follow-up group, the only significantly increased SIR was seen in the BL level group 1.5–1.9 µmol/L, and the P-value for linear trend was 0.17. There was a clear trend in the lung cancer risk over the BL levels in the follow-up groups of 10–19 years and ≥20 year since the last measurement (the P-value for a trend was <0.0001).

**Table 2:** Observed (Obs) number of lung cancer cases (Observed) and standardized lung cancer incidence ratio (SIR) with 95% confidence intervals (CI) by highest personal blood lead and follow-up category. **Bold indicates statistical significance.**

Follow-up ^a^ (years)	Highest personal blood lead value (µmol/L)												Test for trend

	0.0–0.4		0.5–0.9		1.0–1.4		1.5–1.9		2.0–2.9		3.0–7.8		
						
	Obs	SIR (CI)	Obs	SIR (CI)	Obs	SIR (CI)	Obs	SIR (CI)	Obs	SIR (CI)	Obs	SIR (CI)
0–9	10	0.85 (0.41–1.56)	33	0.91 (0.63–1.28)	28	1.38 (0.92–1.99)	16	**2.01 (1.15–3.26)**	8	1.02 (0.44–2.02)	3	0.92 (0.19–2.68)	0.166
10–19	12	0.58 (0.30–1.01)	66	1.10 (0.85–1.40)	30	0.93 (0.63–1.32)	25	**2.01 (1.30–2.97)**	25	**2.06 (1.33–3.04)**	7	1.60 (0.64–3.29)	0.0001
≥20	42	0.91 (0.65–1.23)	163	1.13 (0.96–1.32)	111	**1.43 (1.18–1.72)**	42	**1.41 (1.02–1.90)**	42	**1.67 (1.20–2.26)**	27	**3.10 (2.04–4.51)**	< 0.0001
Overall	65	0.81 (0.62–1.04)	262	1.09 (0.96–1.23)	169	**1.30 (1.11–1.51)**	83	**1.65 (1.32–2.05)**	75	**1.66 (1.31–2.08)**	37	**2.26 (1.59–3.12)**	< 0.0001

a Time since last measurement

The internal analysis, with similar adjustment, yielded stronger associations than the external SIR analysis (table 3). The personal mean BL also fitted slightly better than the highest value. The risk in association with lead was seen in both genders (P-value 0.41 for the interaction term for BL and gender). For most employees the duration of the monitored employment was at least five years. In workers with a very high BL value (average ≥2.0 µmol/L) the duration tended to be shorter than in others. The risk of lung cancer associated strongly with BL levels in the employees with the duration of employment in the monitored tasks being >60 months. However, the risk increased by the duration of employment also by other exposure categories for lead, and there was no indication of interaction in the lung cancer risk between the BL level and duration of employment.

**Table 3:** Internal analyses by highest and mean personal blood lead level and duration of employment in the monitored workplace. Cox regression, hazard ratios (HR) with 95% confidence intervals (CI) adjusted for age, gender, year of the last measurement, socio-economic status in 1975 and occupation- and gender-specific prevalence of regular tobacco smoking. **Bold indicates statistical significance.**

Exposure category	Cases (N)	HR	95% CI
Highest personal blood lead ^a^			
0.0–0.4	65	1.00	reference
0.5–0.9	259	**1.39**	**1.05–1.83**
1.0–1.9	252	**1.81**	**1.37–2.40**
≥2.0	111	**2.28**	**1.67–3.12**
Mean personal blood lead ^a^			
0.0–0.4	84	1.00	reference
0.5–0.9	273	**1.31**	**1.02–1.69**
1.0–1.9	264	**1.93**	**1.49–2.49**
≥2.0	66	**2.58**	**1.85–3.59**
Duration of employment ≥60 months and mean personal blood lead ^a^			
0.0–0.4	65	1.00	reference
0.5–0.9	191	1.16	0.87–1.55
1.0–1.9	173	**1.72**	**1.28–2.31**
≥2.0	32	**2.63**	**1.71–4.05**
Duration of employment 6–59 months and mean personal blood lead ^b^			
0.0–0.4	8	1.00	reference
0.5–0.9	39	1.90	0.87– 4.14
1.0–1.9	39	**2.28**	**1.04–5.02**
≥2.0	14	2.47	0.996– 6.11
Duration of employment <6 months and mean personal blood lead ^c^			
0.0–0.4	2	1.00	reference
0.5–0.9	10	1.50	0.29– 7.83
1.0–1.9	11	2.02	0.39–10.3
≥2.0	8	3.53	0.62–20.1
Duration of employment unknown and mean personal blood lead ^d^			
0.0–0.4	9	1.00	reference
0.5–0.9	33	1.75	0.83–3.71
1.0–1.9	41	**2.82**	**1.34–5.92**
≥2.0	12	**2.52**	**1.03–6.15**

a P for trend <0.0001

b P for trend 0.008

c P for trend 0.13

d P for trend 0.004

The point estimate for the highest BL group (mean personal BL level ≥2.0 µmol/L) was 2.58 (95% CI 1.85–3.59) in the multivariable model without occupational confounders. Adjustment for the studied potential confounders sis not essentially affect the findings (table 4).

**Table 4:** Internal analyses by the studied potential confounding occupational exposures, without and with the grouped mean personal blood lead level. Cox regression, hazard ratios (HR) with 95% confidence intervals (CI) adjusted for age, gender, year of the last measurement and socio-economic status in 1975. Each of the potential occupational confounder was fitted in a set of separate models. **Bold indicates statistical significance.**

Studied potential confounder (unit)	Mean (range)	Lead and daily smoking prevalence not included in the model		Lead and daily smoking prevalence included in the model			
	
		HR for the studied potential confounder		HR for the studied potential confounder		HR for mean blood lead level ≥2.0 µmol/L	
		
		HR	P for trend	HR	P for trend	HR	95% CI
Asbestos [(f/cm^3^)×year×10–1]	0.073 (0–5.4)	**1.37**	**0.03**	1.32	0.09	**2.58**	**1.85–3.59**
Chromium and compounds [(µg/m^3^)×year×10–1]	2.2 (0–35.9)	1.01	0.42	0.99	0.55	**2.60**	**1.86–3.62**
Nickel [(µg/m^3^)×year×10–1]	1.3 (0–28.6)	1.02	0.11	1.00	0.81	**2.57**	**1.84–3.59**
Arsenic [(ug/m^3^)×year×10–1]	0.048 (0–4.7)	1.15	0.14	1.01	0.96	**2.57**	**1.84–3.59**
Cadmium [(µg/m^3^)×year×10–1]	0.051 (0–2.3)	1.37	0.21	1.05	0.86	**2.57**	**1.84–3.59**
Quartz dust [(mg/m^3^)×year×10–1]	0.022 (0–0.83)	**2.82**	**0.001**	1.71	0.12	**2.49**	**1.78–3.48**
Respirable dust [(mg/m^3^)×year×10–1]	1.1 (0–13.8)	**1.04**	**0.007**	1.02	0.22	**2.52**	**1.80–3.52**
Gasoline engine exhaust [(mg/m^3^)×year×10–1]	1.4 (0–34.1)	**0.97**	**0.03**	**0.97**	**0.046**	**2.56**	**1.83–3.56**
Diesel engine exhaust [(mg/m^3^)×year×10–1]	0.017 (0–0.54)	0.23	0.19	0.26	0.22	**2.56**	**1.83–3.57**
Polycyclic aromatic hydrocarbons [(µg/m^3^)×year×10–1]	0.51 (0–28.3)	**1.02**	**0.04**	1.01	0.57	**2.55**	**1.83–3.57**
Benzo(a)pyrene [(µg/m^3^)×year×10–1]	0.040 (0–2.0)	**1.44**	**0.004**	1.19	0.21	**2.51**	**1.80–3.51**

Table 5 presents results by most frequently monitored lead industries (lead battery manufacture, metal foundries, lead smelting). Due to small numbers, the two lowest exposure groups were combined in these analyses. The point estimates of lung cancer risk were seemingly a little higher for the lead battery industry than for other industries. The difference was not statistically significant (P=0.073 for the interaction term between BL and storage battery factories). There were no statistically significant interactions between the other main industry groups and BL level, either (P=0.61 between BL and metal foundries, P=0.71 between BL and the lead smelting; and P=0.27 between BL and other lead industries).

**Table 5:** Internal analyses within lead industries by mean personal blood lead level and potential confounding occupational exposures. Cox regression, hazard ratios (HR) with 95% confidence intervals (CI) adjusted for age, gender, year of the last measurement, socio-economic status in 1975, occupation- and gender-specific prevalence of regular tobacco smoking; and potential confounding occupational exposures. Models for the three lead industries combined was adjusted in addition to exposure to asbestos and benzo(a)pyrene; the model for lead battery factories to asbestos and arsenic; the model for metal foundries to benzo(a)pyrene; and the model for lead smelters to asbestos. **Bold indicates statistical significance.**

Lead industry and duration of employment in the monitored work	Blood lead level (µmol/L)					P for trend

	0.0-0.9 (reference)	1.0-1.9		≥2.0+		
		
	Cases	Cases	HR (95% CI)	Cases	HR (95% CI)	
Employment (months) – industries with most regular measurements	8	39	**2.76 (1.28– 5.97)**	34	**3.42 (1.54–7.63)**	**0.0008**
≥60	2	18	**5.87 (1.30–26)**	11	**10.1 (2.01–51)**	**0.005**
6–59	1	8	3.91 (0.47–33)	10	5.99 (0.65–55)	**0.007**
<6	3	7	2.54 (0.59–11)	3	1.43 (0.22–9.20)	0.59
Unknown	2	6	1.94 (0.38–9.85)	10	2.50 (0.53–12)	0.37
Lead battery factories	4	23	**3.14 (1.07–9.20)**	17	**4.78 (1.55–14.7)**	**0.001**
Metal foundries	2	10	2.38 (0.46–12.2)	7	2.73 (0.48–15.6)	0.48
Lead smelters	2	6	4.20 (0.62–29)	12	3.57 (0.48–27)	0.07
Employment (months) – other industries	349	225	**1.45 (1.24–1.75)**	32	**1.81 (1.26–2.61)**	**<0.0001**
≥60	254	155	**1.45 (1.18–1.78)**	21	**2.16 (1.38–3.38)**	**<0.0001**
6–59	46	31	1.26 (0.79–2.03)	4	0.79 (0.27–2.14)	0.17
<6	9	4	1.01 (0.29–3.54)	5	**4.91 (1.42–17)**	0.12
Unknown	40	35	**1.95 (1.22–3.11)**	2	0.70 (0.17–2.96)	**0.009**

## Discussion

Occupational exposure to lead, measured as BL levels, associated with the lung cancer risk in this study. The increased risk was seen already at rather low exposure levels compared with occupational exposure limit values, and there was no clear threshold value for the increased risk. Particularly high risks were seen in lead-exposed workers with a long duration of employment. The studied occupational carcinogens or tobacco smoking did not explain the risks associated with lead exposure.

The follow-up period was 24 years longer in this study than the initial follow-up published in mid-1990s (9) resulting in 690 incident lung cancers compared to initial 121 cases. The long follow-up time since the collection of the monitoring data was essential for the increased risk estimates for lead. Given the rather short monitoring period compared with the overall employment histories within the workplaces, the long follow-up time reduced potential for bias related to selection of healthy workers in the study. We could also reduce a possible systematic error for a follow-up study due to the incompleteness in the verification of the personal identifiers of the monitored employees in the retrospective data collection phase. The long follow-up time since the monitoring activity caused some limitations too: it was not possible to collect further information from the study subjects or their next-of-kins, or to assess from the available register-based data sources when the exposure to lead had possibly ended or use of lead ended in the workplaces. The lead exposure levels in the monitored workplaces had continuously decreased since 1960s and early 1970s (13, 19). After the monitoring period of the current study many workplaces had been closed down or stopped using lead or discontinued the monitoring activity due to other reasons (20). Lead exposure from environmental sources also decreased, particularly after reductions of lead content in gasoline and prohibition use of leaded gasoline in 1994. Also, possibilities for longer exposure assessment periods <2 µmol/L were reduced because the instructions to continued monitoring were directed mainly to preventing very high-level exposures.

In the initial follow-up study of this cohort, information on exposure histories and personal tobacco smoking were assessed in a nested case–control study (9, 13). Tobacco smoking habits and histories as well as detailed employment histories (employers, workplaces, occupations, descriptions of work tasks) were assessed from the study subjects or their next-of-kins with help of a questionnaire, and assessments of exposure histories were compiled based on the information on the collected employment histories, monitoring, and industrial hygienic data. Potential occupational confounders or risk modifiers were common in many tasks, eg, in metal foundries, lead smelting, and car repair. However, probability of exposure to confounders was assessed as low among heavily exposed workers in storage battery manufacturing. Efforts were made in the current study to assess potential occupational confounders using data on occupational groups from historical census records, combined with FINJEM; as well as assess duration of employment using the pension center records. We believe that these register-based data sources were very valuable for the long-term follow-up. Except the short-term workers, contrasts of lead exposure by average BL were remarkable even based on few or a single individual-level measurements, if the duration of the employment period was long [see (21)]. The monitored employees were from a variety of occupational activities with variable patterns in potential confounders. We did not see any remarkable confounding by the studied occupational carcinogens, and there was no clear interaction between BL and the industry for the lung cancer risk. Still, we cannot completely rule out some residual confounding, due to use of group-level job-exposure-matrix data.

Tobacco smoking did not materially confound the risk associated with lead in the initial case–control study (9, 13). The unadjusted odds ratio (OR) for the highest personal BL category ≥2.0 µmol/l was 1.2 (95% CI 0.4–4.1), and the OR adjusted for tobacco smoking (based on smoking history data) and vital status was 1.5 (95% CI 0.4–5.8), respectively (9). Alcohol consumption did not play a role in the lung cancer risk in that study. Information on other lifestyles, eg, diet or body-mass index, was not available. It had also been demonstrated that tobacco smoking did not affect the BL levels in the general Finnish population; the average BL 0.62 μmol/L in smokers and 0.58 μmol/L in non-smokers (14). In the current study, control for tobacco smoking could be done using occupational- and sex-specific daily smoking prevalence. Even though the smoking prevalence associated with about an 11-fold statistically highly significant increase in the lung cancer risk in the age- and gender-adjusted model, it did not confound the result observed for lead. About 75% of cohort members were blue-collar workers and final results were adjusted also for the SES. Still, in the current study we cannot rule out possible residual confounding by tobacco smoking particularly in the very low BL levels, where also excess risks of lung cancer are rather small compared to the heavy occupational exposure.

Adjustment for the SES might have produced a risk of over adjustment. However, the comparable risk estimate for the mean personal BL ≥2.0 µmol/L as shown in table 3 (HR 2.58, 95% CI 1.85–3.59) was 2.62 (95% CI 1.88–3.65) without adjustment for the SES. The CI did not become wider, and we therefore consider the risk of over adjustment small. Moreover, adjustment for SES was justified as a surrogate of unmeasured lifestyle-related factors.

In the previously reported pooled analysis of employees biologically monitored for BL from Finland, UK and US there were statistically significant positive trends using the log of each worker’s maximum BL value for mortality from lung cancer, chronic obstructive pulmonary disease (COPD), stroke and heart disease, while borderline significant trend were found for mortality from bladder cancer, brain cancer and larynx cancer (11). There was a significant interaction for lung cancer between the countries, however (UK positive trend, P=0.14; USA/Finland positive trends, P<0.0001). A more recent analysis of cancer incidence in the combined cohort from Finland and the UK also suggested associations with lung cancer. In the separate analysis, the risk was confined mainly to the Finnish cohort and only suggestive association was seen in the UK cohort (12). Because in the UK cohort the maximum personal BL was <10 µg/dL (about 0.48 µmol/L) for only 1% of the employees, comparisons in that study could not be done using a reference category with such a low BL as here.

Follow-up studies of occupationally exposed employees with BL data have been published also from Australia (3) and South Korea (4). In the Gwini et al study (3) there was a small increase in the lung cancer risk in the overall cohort and no risk in a small subgroup of employees in the high BL category (>30 µg/dL; corresponding roughly to >1.45 µmol/L) compared with the general population. There were limitations in the quality and nature of the original cohort records, which according to the authors may have influenced the outcome ascertainment. Kim et al (4) reported increased risk of lung cancer mortality among women but not men, based yet on small numbers of exposed cases.

In the study by Liao et al (22), an excess risk of lung cancer after controlling for smoking was found for men but not women. Wynant et al (23) found no elevation of lung cancer in exposed workers in a population-based case–control study in Montreal. No personal biological measurements were available in that study.

BL from environmental exposures correlated with lung cancer mortality after an adjustment for cigarettes smoked per day, alcohol consumption, poverty income ratio, urban-rural residence status, body mass index, gender and few other potential confounders related mainly to lifestyle (6). The mean BL level in the study was as low as 4.4 µg/dL (about 0.2 µmol/L). Information on exposure to other environmental pollutants, such as other metals or engine exhausts, was not available. Rhee et al (24) reported increased risks of lung cancer mortality for BL values mainly from environmental exposures. Increased BL levels studied ranged from <10 to ≥20 µg/dL (about 0.48–0.97 µmol/L), and they associated with smoking status and gender. The lung cancer mortality risk was associated with BL among women but not men, after an adjustment for smoking status, median pack-years, serum cotinine and environmental tobacco smoke exposure, and several variables on population characteristics. The association between BL and lung cancer was restricted to current and former smokers; lung cancer risk was unusually low in lead-exposed non-smokers. Therefore, the authors did not rule out residual confounding because of smoking. Effect modification between lead and tobacco smoking was seemingly also possible, but no formal analyses were provided.

The current study documents excess risk of lung cancer in workers occupationally exposed to inorganic lead. Compared to the initial study (9), the follow-up time is now reasonably long and number of cases large. Unlike follow-up of two cohorts (12), we could consider occupation- and gender-specific regular tobacco smoking and exposure to main occupational risk factors. Moreover, the Finnish data included relatively big internal low-exposure reference group. Thus, our study adds credibility into the findings of the earlier cohort studies.

### Concluding remarks

The current study supports the findings from earlier studies suggesting that exposure to lead increases risk of lung cancer. The risk was observed with even rather low BL levels compared with the occupational exposure standards of the time of the study. The previous studies have reported increased risks of lead also for some primary sites of cancer other than lung, such as esophagus and brain cancers as well as lymphoma, and it would be important to study effects of lead also with those primary sites with help of information on exposure histories and potential confounders.

### Conflicts of interest statement

The authors declare no conflicts of interest.

### Funding

The study has been financially supported by the Finnish Work Environment Fund (Grant No. 113246). The funder did not have any role in the (i) study design, (ii) the collection, analysis and interpretation of the data, (iii) the writing of the report, and (iv) the decision to submit the paper for publication.

## Supplementary material

Supplementary material

## References

[1] International Agency for Research on Cancer (IARC) (2006). Inorganic and Organic Lead Compounds.

[2] Ward EM, Schulte PA, Straif K, Hopf NB, Caldwell JC, Carreón T (2010). Research recommendations for selected IARC-classified agents. Environ Health Perspect.

[3] Gwini S, MacFarlane E, Del Monaco A, McLean D, Pisaniello D, Benke GP (2012). Cancer incidence, mortality, and blood lead levels among workers exposed to inorganic lead. Ann Epidemiol.

[4] Kim MG, Ryoo JH, Chang SJ, Kim CB, Park JK, Koh SB (2015). Blood Lead Levels and Cause-Specific Mortality of Inorganic Lead-Exposed Workers in South Korea. PLoS One.

[5] McElvenny DM, Miller BG, MacCalman LA, Sleeuwenhoek A, van Tongeren M, Shepherd K (2015). Mortality of a cohort of workers in Great Britain with blood lead measurements. Occup Environ Med.

[6] Cheung MR (2013). Blood lead concentration correlates with all cause, all cancer and lung cancer mortality in adults:a population based study. Asian Pac J Cancer Prev.

[7] Taskinen H, Hogstedt C, Reuterwall C (1988). Spontaneous abortions among women occupationally exposed to lead. Progress in Occupational Epidemiology.

[8] Lindbohm ML, Sallmén M, Anttila A, Taskinen H, Hemminki K (1991). Paternal occupational lead exposure and spontaneous abortion. Scand J Work Environ Health.

[9] Anttila A, Heikkilä P, Pukkala E, Nykyri E, Kauppinen T, Hernberg S (1995). Excess lung cancer among workers exposed to lead. Scand J Work Environ Health.

[10] Anttila A, Heikkilä P, Nykyri E, Kauppinen T, Pukkala E, Hernberg S (1996). Risk of nervous system cancer among workers exposed to lead. J Occup Environ Med.

[11] Steenland K, Barry V, Anttila A, Sallmén M, McElvenny D, Todd AC (2017). A cohort mortality study of lead-exposed workers in the USA, Finland and the UK. Occup Environ Med.

[12] Steenland K, Barry V, Anttila A, Sallmen M, Mueller W, Ritchie P (2019). Cancer incidence among workers with blood lead measurements in two countries. Occup Environ Med.

[13] Anttila A (1994). Occupational risk of lead and risk of cancer. Acta Universitatis Tamperensis Ser A Vol 417.

[14] Nordman H (1975). Environmental lead exposure in Finland. A study on selected population groups. Academic dissertation.

[15] Pukkala E, Guo J, Kyyrönen P, Lindbohm ML, Sallmén M, Kauppinen T (2005). National job-exposure matrix in analyses of census-based estimates of occupational cancer risk. Scand J Work Environ Health.

[16] Finnish Job Exposure Matrix (FINJEM) (2016). Excel version 2016. Finnish Institute of Occupational Health (FIOH).

[17] Lahelma E, Rahkonen O, Berg MA, Helakorp S, Prättälä R, Puska P (1997). Changes in health status and health behavior among Finnish adults 1978-1993. Scand J Work Environ Health.

[18] Maldonado G, Greenland S (1993). Simulation study of confounder-selection strategies. Am J Epidemiol.

[19] Hernberg S, Tola S (1979). The battle against occupational lead poisoning in Finland. Experiences during the 15-year period 1964--1978. Scand J Work Environ Health.

[20] Anttila A, Jaakkola J, Tossavainen A, Vainio H (1992). Työperäinen kemikaalialtistuminen Suomessa. Altisteet työssän:o 34.

[21] Barbosa F Jr, Tanus-Santos JE, Gerlach RF, Parsons PJ (2005). A critical review of biomarkers used for monitoring human exposure to lead:advantages, limitations, and future needs. Environ Health Perspect.

[22] Liao LM, Friesen MC, Xiang YB, Cai H, Koh DH, Ji BT (2016). Occupational lead exposure and associations with selected cancers:the Shanghai Men's and Women's Health Study Cohorts. Environ Health Perspect.

[23] Wynant W, Siemiatycki J, Parent MÉ, Rousseau MC (2013). Occupational exposure to lead and lung cancer:results from two case-control studies in Montreal, Canada. Occup Environ Med.

[24] Rhee J, Graubard BI, Purdue MP (2021). Blood lead levels and lung cancer mortality:an updated analysis of NHANES II and III. Cancer Med.

