# Supplementary material for: Lung cancer incidence among workers biologically monitored for occupational exposure to lead: a cohort study
Source: Scand J Work Environ Health. 2022 Oct 1;48(7):540–8. doi: 10.5271/sjweh.4046 (PMC10539111; doi:10.5271/sjweh.4046)
Supplement: Supplementary material [file SJWEH-48-540-S001.pdf]

Lung cancer incidence among workers biologically monitored for occupational exposure to lead: a cohort study [1]

by Ahti Anttila, PhD,[2] Sanni Uuksulainen, MSc, Matti Rantanen, MSc, Markku Sallmén, PhD

1. Supplementary material
2. Correspondence to: Ahti Anttila, Finnish Cancer Registry, Unioninkatu 22, 00130 Helsinki, Finland. [E-mail: ahti.anttila@cancer.fi]

Supplementary table S1. Definitions of FINJEM agents (unit of level, definition of unit and assessment threshold) used as occupational confounders.

**FINJEM agent**	**Unit of level**	**Definition of unit**	**Assessment threshold**
Asbestos	f/ cm³	Fibers (length over 5µm, diameter below 3µm, aspect ratio at least 3:1) of asbestos in cubic centimeter of workroom air.	Possibly at least 5% of the occupation exposed to an annual mean level of 0.01 f/cm³ of the agent at any time in 1945-95.
Chromium	µg/m³	Microgram of agent in cubic meter of workroom air.	Possibly at least 5% of the occupation exposed to an annual mean level of 0.5 µg/m³ of the agent at any time in 1945-95.
Nickel	µg/m³	Microgram of agent in cubic meter of workroom air.	Possibly at least 5% of the occupation exposed to an annual mean level of 1 µg/m³ of the agent at any time in 1945-95.
Arsenic	µg/m³	Microgram of agent in cubic meter of workroom air.	Possibly at least 5% of the occupation exposed to an annual mean level of 0.5 µg/m³ of the agent at any time in 1945-95.
Cadmium	µg/m³	Microgram of agent in cubic meter of workroom air	Possibly at least 5% of the occupation exposed to an annual mean level of 0.5 µg/m³ of the agent at any time in 1945-95.
Quartz dust	mg/m³	Milligram of agent in cubic meter of workroom air.	Possibly at least 5% of the occupation exposed to an annual mean level of 0.02 mg/m³ of the agent at any time in 1945-95.
Respirable dust	mg/ m³	Milligram of agent in cubic meter of workroom air.	Possibly at least 5% of the occupation exposed to an annual mean level of 0.02 mg/m³ of the agent at any time in 1945-95.
Gasoline engine exhaust	mg/m³	Milligrams of carbon monoxide in cubic meter of workroom air.	Possibly at least 5% of persons in the occupation exposed at work to an annual mean level of at least 2 mg/ m³ of carbon monoxide at some time in 1945-95.
Diesel engine exhaust	mg/m³	Milligrams of nitrogen dioxide in cubic meter of workroom air.	Possibly at least 5% of persons in the occupation exposed at work to an annual mean level of at least 0.05 mg/ m³ of nitrogen dioxide at some time in 1945-95.
Polycyclic aromatic hydrocarbons	µg/m³	Microgram of all PAH-compounds in cubic meter of workroom air.	Possibly at least 5% of the occupation exposed to an annual mean level of 0.1 µg/m³ of the agent at any time in 1945-95.
Benzo(a)pyrene	µg/m³	Microgram of benzo(a)pyrene in cubic meter of workroom air.	Possibly at least 5% of the occupation exposed to an annual mean level of 0.005 µg/m³ of the agent at any time in 1945-95.

Supplementary table S2. Internal analyses by the studied potential confounding occupational exposures, without and with the grouped mean personal blood lead level. For blood lead level, results only for the highest category (2.0+ µmol/L) is shown in the table in the right, compared with the lowest BL group. Cox regression, hazard ratios (HR) with 95% confidence intervals (CI) adjusted for age, gender, year of the last measurement and socio-economic status in 1975. Each of the potential occupational confounder was fitted in a set of separate models.

		Lead and daily smoking prevalence not included in the model	Lead and daily smoking prevalence included in the model			
		Studied potential confounder	Studied potential confounder		Mean blood lead level 2.0+ µmol/l	
Studied potential counfounder, unit and exposure category	N cases	HR and trend test	HR and trend test	CI	HR	CI
Asbestos ((f/cm^3)*year)						
[0,0.001)	235	1.00	1.00			
[0.001,2)	328	0.95	0.91	0.77-1.09		
[2,Inf)	124	1.23	1.15	0.91-1.45		
Continuous (*P for trend*)		***0.03***	*0.09*			
					**2.58**	**1.85-3.59**
Chromium and its compounds ((µg/m^3)*year)						
[0,0.001)	242	1.00	1.00			
[0.001,100)	404	1.14	1.05	0.88-1.24		
[100,Inf)	41	1.10	0.90	0.63-1.28		
Continuous (*P for trend*)		*0.42*	*0.55*			
					**2.60**	**1.86-3.62**
Nickel ((µg/m^3)*year)						
[0,0.001)	305	1.00	1.00			
[0.001,40)	300	1.18	1.09	0.93-1.29		
[40,Inf)	82	1.09	0.94	0.72-1.21		
Continuous (*P for trend*)		*0.11*	*0.81*			
					**2.57**	**1.84-3.59**
Arsenic ((µg/m^3)*year)						
[0,0.001)	567	1.00	1.00			
[0.001,1)	53	1.24	1.25	0.94-1.66		
[1,Inf)	67	1.54	1.26	0.97-1.64		
Continuous (*P for trend*)		*0.14*	*0.96*			
					**2.57**	**1.84-3.59**
Cadmium ((µg/m^3)*year)						
[0,0.001)	299	1.00	1.00			
[0.001,2)	318	0.94	0.89	0.76-1.05		
[2,Inf)	70	1.26	1.06	0.80-1.39		
Continuous (*P for trend*)		*0.21*	*0.85*			
					**2.57**	**1.84-3.59**
Quartz dust ((mg/m3)*year)						
[0,0.001)	530	1.00	1.00			

[0.001,1)	81	1.39	**1.33**	**1.05-1.68**		
[1,Inf)	76	1.57	**1.29**	**1.00-1.66**		
Continuous (*P for trend*)		***0.001***	*0.12*			
					**2.49**	**1.78-3.48**
Respirable dust ((mg/m3)*year)						
[0,0.001)	140	1.00	1.00			
[0.001,40)	447	1.24	1.18	0.97-1.44		
[40,Inf)	100	1.33	1.17	0.88-1.54		
Continuous (*P for trend*)		***0.007***	*0.22*			
					**2.52**	**1.80-3.52**
Gasoline engine exhaust ((mg/m³)*year)						
[0,0.001)	479	1.00	1.00			
[0.001,100)	183	1.06	1.07	0.89-1.27		
[100,Inf)	25	0.75	0.75	0.50-1.13		
Continuous (*P for trend*)		***0.03***	***0.05***			
					**2.56**	**1.83-3.56**
Diesel engine exhaust ((mg/m³)*year)						
[0,0.001)	478	1.00	1.00			
[0.001,0.5)	142	1.18	1.18	0.98-1.43		
[0.5,Inf)	67	0.77	0.78	0.60-1.01		
Continuous (*P for trend*)		*0.19*	*0.22*			
					**2.56**	**1.83-3.57**
Polycyclic aromatic hydrocar-bons ((µg/m³)*year)						
[0,0.001)	339	1.00	1.00			
[0.001,20)	280	0.93	0.88	0.74-1.04		
[20,Inf)	68	1.49	1.17	0.88-1.55		
Continuous (*P for trend*)		***0.04***	*0.57*			
					**2.55**	**1.83-3.57**
Benzo(a)pyrene ((µg/m³)*year)						
[0,0.001)	411	1.00	1.00			
[0.001,1)	211	0.96	0.92	0.78-1.09		
[1,Inf)	65	1.54	1.22	0.92-1.61		
Continuous (P for trend)		***0.004***	*0.21*			
					**2.51**	**1.80-3.51**
